# Evaluation of provincial forest ecological security and analysis of the driving factors in China via the GWR model

**DOI:** 10.1038/s41598-024-65052-x

**Published:** 2024-06-21

**Authors:** Longbo Ma, Binyu Yang, Yan Feng, Liyu Ju

**Affiliations:** 1 School of Economics and Management, Qingdao Agricultural University, Qingdao, 266109 China; 2 School of Business, Liaocheng University, Liaocheng, 252000 China

**Keywords:** Ecology, Ecology, Environmental sciences, Environmental social sciences

## Abstract

Economic and social factors pose an enormous threat to forest ecological security, which in turn affects the development of ecological civilization. This paper applies a geographically weighted regression model to study the impact of urbanization, industrial structure, and foreign direct investment on forest ecological security of 30 provinces in 2013 and 2018 and reveals the spatial differences of various influencing factors in different provinces. First, the overall forest ecological security in China is at an average level, with slight improvement. Second, urbanization has the greatest impact on forest ecological security in all provinces, which shows a rising trend. Third, the positive impact of industrial structure on forest ecological security has improved. Fourth, in 2013, FDI (foreign direct investment) had a negative correlation with forest ecological security. In 2018, FDI had a positive impact on the forest ecological security index of 16 provinces, while it had a negative correlation in other provinces. Therefore, it is necessary to formulate a coordinated security improvement plan for neighboring provinces. The government should promote rural revitalization, plan the layout of cities rationally, increase the proportion of tertiary industry, appropriately raise access barriers to foreign investment and strengthen environmental supervision over existing foreign-invested enterprises.

## Introduction

Forests are the most important and biologically rich ecosystems on land. A healthy forest ecosystem can effectively prevent land degradation, soil desertification and natural disasters and provide air purification, climate regulation, soil and water conservation and other ecosystem services, which plays an important role in global ecological sustainability. However, with the rapid advance of urbanization and industrialization, human activities have strongly interfered with the evolution of natural ecosystems. Extensive open utilization has led to a sharp decline in forest area and forest cover. A series of ecological and environmental problems appears, such as air pollution, desertification, and loss of species diversity, which poses a great threat to forest health and sustainability. To describe and measure the health status, function and sustainability level of forest ecosystem, the concept of forest ecological security has been proposed by scholars in recent years and has attracted extensive attention of relevant scholars. Costanza (2012)^1^ argues that forest ecological security should focus on the structure and function of forest ecosystems to achieve their health, integrity and sustainability, while human interference and maintenance behaviors should also be reflected in it^2^. In summary, forest ecological security can be defined as a state in which forest ecosystems in a country or region can achieve self-regulation, self-restoration, and maintenance of sustainability, complexity, restorability and serviceability after being impacted by external environment and human socioeconomic activities within a certain spatial and temporal range^3^.

As the third-largest country in the world in terms of land area, China has approximately 220 million hectares of forest, accounting for approximately 5.4% of the global forest area. However, long-term unreasonable human activities have destroyed the forest ecosystem and seriously affected the sustainable environmental development of the country. Moreover, differences in geographical conditions and socioeconomic development cause significant differences in the resource endowment and status of forest ecosystems in different regions. The impacts of many ecological restoration projects and their effects on the process of implementation also differ. Therefore, to promote ecologically sustainable development, it is urgent to research the state of forest ecological security and its driving factors to facilitate the formulation and implementation of differentiated policies.

In recent years, scholars have carried out much research on forest ecological security measurement and its driving factors. In terms of forest ecological security measurement, according to data from statistical yearbooks and statistical websites, the entropy weight method, expert method, fuzzy object element method, K-means clustering method, variable weight theory, G1 method, principal component analysis, object element analysis, fuzzy evaluation, Moran′s I index, spatial convergence model, improved TOPSIS model, hierarchical dynamic attitude model, Markov chain model, GIS spatial analysis method and barrier degree model are adopted. It concludes that the status of forest ecological security greatly varies among provinces (cities and autonomous regions) and regions in provinces (cities and autonomous regions) in China and that the phenomenon of polarization occurs^4–11^. Studies on driving factors of forest ecological security have focused mostly on aspects such as demographic and economic. The results of relevant studies conducted by using Tobit model and structural equation model show that rural labor outflow, primary forestry industry development and tertiary forestry industry development have positive effects on enhancing forest ecological security^12–14^. However, there is an inverted U-shaped relationship between urbanization and the forest ecological security index with temporal and geographical variability^15^. Some scholars believe that urbanization and the development of secondary industry have a negative impact on forest ecological security^16,17^, and foreign direct investment has a U-shaped relationship with forest ecological security^18^, however, its relationship with the pollution status on the environment has not led to a consistent conclusion^19,20^.

In summary, there are abundant studies on the spatial and temporal evolution of forest ecological security and its driving factors, laying a solid foundation for the smooth development of this area. Although existing studies have explored various aspects of the forest ecological security index system. Additional county- and city-level evaluations of a single province have been conducted for a single province and forest situation^5,6,21^. Literatures on the evaluation of forest ecological security at the provincial level in China^4,7^ are limited. However, it is also important to pay attention to the following two aspects: first, the impact of the driving factors on forest ecological security is not linear, and there is spatial variability; second, forest externalities and the forest ecological environment are mutually influential among various provinces, cities and autonomous regions, and spatial dependence occurs.

With the implementation of China's forestry policies, such as land greening and ecological protection, the national and forest conditions in China have changed dramatically^22^. Therefore, this study has three innovative aspects and objectives . The first is to improve the traditional PSR (pressure-state-response) model, establish a forest ecological security index system based on forestry census data, and enrich the existing research. The second is comprehensively evaluate the status of forest ecological security in China, describe its spatiotemporal distribution, and pay attention to its spatial dependence. The third is to use a geographically weighted regression model to study in depth the spatial differences in forest ecological security in terms of urbanization, industrial structure and foreign direct investment. The spatial differences in forest ecological security among 30 provinces, cities and autonomous regions in China are analyzed from the perspective of spatial effects, and the problem that the impact of driving factors on forest ecological security is not a single linear relationship is addressed. In this way, we can theoretically fill the gaps in existing research, expand the application scope of PSR model and geographically weighted regression model, and practically guide the formulation of differentiated policies for the country and regions to help accomplish the goal of protecting forest ecosystems.

## Materials and methods

### Measurement of the forest ecological security level

#### Establishment of the forest ecological security index system

The PSR model is commonly used for evaluating ecosystem security and is divided into three types of indicators: pressure indicators, status indicators, and response indicators. Among them, pressure indicators aim to describe the role of economic and social factors in the ecological environment; state indicators are used to indicate the state and changes in the ecological environment in a certain period; and response indicators refer to negative impacts on the ecological environment through a series of prevention, mitigation, prevention and remediation of economic and social activities by human beings^23^. The PSR model is currently respected by many scholars at home and abroad and is used in the study of the ecological environment system. In the study of the evaluation and driving factors of forest ecological security, the PSR framework is mostly used to construct an index system for evaluating forest ecological security, which classifies indicators into pressure, state, and response indicators; this classification helps to determine the interactions between them^24^. In the PSR model, pressure (P) refers to the harmful interference caused by economic and social factors on forest ecosystems, while response (R) refers to the maintenance made by economic and social factors on forest ecosystems. Both of them refer to "pressure" in this paper. The former is "positive pressure", while the latter is "negative pressure". Therefore, in this paper, we calculated the forest ecological security pressure index, including positive pressure and negative pressure, but didn’t calculate the response index separately.

The index system is established using the PSR model. Based on existing studies^3–6^ and by the expert meeting method, 31 experts in forestry, ecology, and forestry economics were consulted. The selected indicators were deleted and adjusted, and 10 indicators were ultimately identified to construct the forest ecological security index system. First, in terms of forest status indicators, mainly from the two aspects of quantity and quality, four indicators are selected: the rate of forest cover, percentage of forestland area, forest accumulation per unit area, and percentage of natural forests. Among them, the higher the rate of forest cover and percentage of forestland area are, the greater the forest accumulation per unit area is. This indicates that the quantity of forest is greater, the higher the percentage of natural forests is, the better the forest quality is, and the greater the quantity of forest is, which is conducive to improving the forest ecological security level. Thus, the above four indicators are all positive. Second, three indicators are selected to explain forest pressure: population density, energy consumption per unit area, and sulfur dioxide emission intensity. A higher population density is associated with greater energy consumption per unit area. A higher sulfur dioxide emission intensity indicates that the pressure generated by human activities on forests is greater and will adversely affect forest ecological security. Thus, all three indicators are negative. Finally, as for forest response indicators, three indicators are selected: the proportion of nature reserves, the intensity of government forestry investment and the annual afforestation rate. A higher proportion of nature reserves is associated with a greater intensity of government forestry investment. A higher annual afforestation ratio indicates that human society attaches more importance to forest protection, which is conducive to improving forest ecological security. Thus, the above three indicators are all positive indicators (Table 1).

**Table 1:** Index system of forest ecological security.

Structure	Substructure	Index name (nature)	Formula
Forest status indicators	Quantity indicators	Rate of forest cover (+^1^)	Forest area/land area × 100%
		Percentage of forestland area (+)	Forestland area/national land area × 100%
		Forest accumulation per unit area (+)	Forest stock/Forest area
	Quality indicators	Percentage of natural forests (+)	Natural forest area/forest area × 100%
Forest pressure indicators	General pressure	Population density (−^2^)	Year-end population/Land area
		energy consumption per unit area (−)	Energy consumption/Land area
		Sulfur dioxide emissions	Sulfur dioxide emissions/Land area
Forest response indicators	Maintenance indicators	Proportion of nature reserves (+)	Nature Reserve Area/Land area × 100%
		Government forestry investment intensity (+)	Amount of government forestry investment/GDP
		Annual afforestation rate (+)	Annual afforestation area/Forest area × 100%

1 is a positive indicator, and 2 is an inverse indicator.

#### Calculation method of the forest ecological security index

This paper calculates the forest ecological security index based on the entropy weight method^25^. The entropy weight method is based on the basic principle of information theory. The degree of order of a system can be divided into two states, order and disorder. The degree of order is expressed by information, while the degree of disorder is measured by entropy. The smaller the entropy value is, the greater the amount of information provided by the index is. The entropy weight method has the advantage of assigning objective weights, and it can effectively avoid the subjective influence of weight setting.

1. Entropy method weights

① Assuming that there are m samples and n indicators, the judgment matrix is constructed.

1 $$\text{X}=({\text{x}}_{\text{ij}}{)}_{\text{m}\times \text{n}}(\text{i}=\text{1,2},\cdots ,\text{m};\text{j}=\text{1,2},\cdots ,\text{n})$$

②Standardize the judgment matrix indicator data.

The standardized equation for the forward and inverse indicators of the forest status index is

2 $${\text{y}}_{\text{ij}}=\frac{{\text{x}}_{\text{ij}}-{\text{x}}_{\text{min}}}{{\text{x}}_{\text{max}}-{\text{x}}_{\text{min}}}$$

The standardized equation for the inverse indicators of the forest status index and the positive indicators of the forest pressure index is

3 $${\text{y}}_{\text{ij}}=\frac{{\text{x}}_{\text{max}}-{\text{x}}_{\text{ij}}}{{\text{x}}_{\text{max}}-{\text{x}}_{\text{min}}}$$

${\text{y}}_{\text{ij}}$ is the standardized data of the index, and I and j are the maximum and minimum values of the index, respectively.

③ Calculate the information entropy of indicator j.

4 $${\text{H}}_{{\rm j}}=-\frac{\underset{i=1}{\sum^{n}}{\text{Y}}_{{\rm ij}}{\text{lnY}}_{{\rm ij}}}{\text{lnn}}$$

5 $$ {\text{Y}}_{{{\text{ij}}}} = \frac{{{\text{y}}_{{{\text{ij}}}} }}{{\mathop {\mathop \sum \limits^{n} }\limits_{i = 1} {\text{y}}_{{{\text{ij}}}} }},\quad {\text{i}} = 1,2, \ldots ,{\text{n}} $$

${\text{H}}_{\text{j}}$ is the information entropy of indicator j.

④ Calculate the weight of indicator j.

6 $${\text{w}}_{\text{j}}=\frac{1-{\text{H}}_{\text{j}}}{\underset{j=1}{\sum^{n}}(1-{\text{H}}_{\text{j}})}$$

${\text{w}}_{\text{j}}$ is the weight of indicator j.

The Forest Ecological Security Status Index reflects the health degree of the forest system itself and is calculated as

7 $$Z=\sum_{\text{j}=1}^{{\rm n}}{\text{w}}_{{\rm j}}{\text{y}}_{{\rm ij}}$$

Z is the forest ecological security status index, ${\text{y}}_{\text{ij}}$ is the standardized data of the status index, and ${\text{w}}_{\text{j}}$ is the weight of indicator j.

The FSI reflects the magnitude of the combined socioeconomic development pressure on the forest system and the maintenance of the forest ecology by human activities and is calculated as follows:

8 $$\text{Y}=\sum_{{\rm j}=1}^{{\rm n}}{\text{w}}_{{\rm j}}{\text{y}}_{{\rm ij}}$$

Y is the forest ecological security pressure index, ${\text{y}}_{\text{ij}}$ is the standardized data of pressure indicators, and ${\text{w}}_{\text{j}}$ is the weight of indicator j.

3. Forest Ecological Security Index

The forest ecological security status index and the forest ecological security pressure index are geometrically averaged to obtain the forest ecological security composite index, and the formula is

9 $$ {\text{ESI}} = \sqrt {{\text{Z}} \times \left( {1 - {\text{Y}}} \right)} $$

ESI is the forest ecological security index. Z is the forest ecological security status index. Y is the forest ecological security pressure index.

### Research methods for spatial clustering patterns of forest ecological security in China

This article uses the local correlation index to reflect the spatial clustering pattern of forest ecological security in China. The local correlation index Getis-Ord GI* statistic can identify clusters of higher indicator values (hot spots) and clusters of lower indicator values (cold spots) . It is commonly used to analyze the degree of aggregation of observations in local space. The formula is^26^:

10 $$ G_{i}^{*} \left( d \right) = {\raise0.7ex\hbox{${\mathop {\mathop \sum \limits^{n} }\limits_{{j = 1}} w_{{ij}} (d)x_{j} }$} \!\mathord{\left/ {\vphantom {{\mathop {\mathop \sum \limits^{n} }\limits_{{j = 1}} w_{{ij}} (d)x_{j} } {\mathop {\mathop \sum \limits^{n} }\limits_{{j = 1}} x_{j} }}}\right.\kern-\nulldelimiterspace} \!\lower0.7ex\hbox{${\mathop {\mathop \sum \limits^{n} }\limits_{{j = 1}} x_{j} }$}} $$

${G}_{i}^{*}\left(d\right)$ is standardized, i.e., $ZG_{i}^{*} = \frac{{G_{i}^{*} - E(G_{i}^{*}}}{{\sqrt {Var\left( {G_{i}^{*} } \right)} }}$, where $EG_{i}^{*}$ is the expected value of ${G}_{i}^{*}$, $Var\left({G}_{i}^{*}\right)$ is the variance of ${G}_{i}^{*}$, and ${w}_{ij}(d)$ is the spatial weight. A positive and significant $ZG_{i}^{*}$ indicates that the value of position i and the surrounding area are relatively high (higher than the mean), indicating a high-level clustering area (hot spot area). In contrast, if the value is negative and significant, the value around position i is lower than the mean and correspondingly represents a low-level agglomeration area (cold spot area).

### Analysis of the drivers of forest ecological security

#### Selection of the driving factors of forest ecological security

The forest ecological security index reflects only forest ecological security of a region. Its driving factors are the focus of the paper. Only by identifying its driving factors can we improve the status of forest ecological security. According to previous studies^17,18,20,27^, there are many factors affecting forest ecosystem security, including demographic factors, technology level, location endowment, urbanization process, industrial structure and foreign direct investment. In this paper, the following two aspects are considered when selecting the driving factors. On the one hand, this paper examines the spatial differences in interprovincial forest ecosystem security in China from the perspective of industrial structure upgrading and urbanization. We also examine whether there is a "pollution paradise". On the other hand, the GWR model requires that multiple variables should not have multicollinearity and are prone to multicollinearity. Based on the above analysis, this paper selects urbanization level, industrial structure and foreign direct investment as independent variables to investigate the spatial differences among them on forest ecological security (Table 2).

**Table 2:** Index system for driving factors of forest ecological security in China.

Variable	Indicator calculation
Urbanization level	Urban population/regional permanent population
Industrial structure	Value added of the tertiary industry/Value added of the secondary industry
Foreign direct investment	Foreign direct investment amount

#### Model construction of the driving factors of forest ecological security

Geographic information systems (GISs) are also widely used to analyze the spatiotemporal evolution of forest ecological security^28,29^. AMOS^13^, OLS, SEM^17^ and other methods are mostly used for the analysis of factors influencing forest ecological security. However, the models represented by the least squares (OLS) model are all linear nonspatial regression models that analyze the regression of independent variables on dependent variables in the overall region without introducing spatial distance weights. Thus, they cann’t effectively reflect the regional influence of influencing factors on the forest ecological security index in different regions. The geographically weighted regression model can better reveal spatial differences at different locations. Thus, when studying the spatial relationship between forest ecological security and its influencing factors, the geographically weighted regression model is chosen instead of the OLS model to increase the reliability of the spatial scale. The model is as follows:

11 $$ {\text{y}}_{{\text{i}}} = \beta_{0} \left( {{\text{u}}_{{\text{i}}} {,}\;{\text{v}}_{{\text{i}}} } \right) + \mathop \sum \limits_{{{\text{k}} = 1}}^{{\text{p}}} \beta_{{\text{k}}} \left( {{\text{u}}_{{\text{i}}} {,}\;{\text{v}}_{{\text{i}}} } \right){\text{x}}_{{{\text{ik}}}} + \varepsilon_{{\text{i}}} \quad {\text{i}} = 1,2, \ldots ,{\text{n}} $$

${\text{y}}_{\text{i}}$ is an n × 1-dimensional explanatory variable. This paper refers to the forest eco-logical security index. (${\text{u}}_{\text{i}}$, ${\text{v}}_{\text{i}}$) is the coordinate of observation point i. βk (${\text{u}}_{\text{i}}$, ${\text{v}}_{\text{i}}$) is the kth regression coefficient on observation point i. And ${\upvarepsilon }_{\text{i}}$ is the residual of the algorithm.

The core of the geographically weighted regression model is the spatial weight matrix. The selection of an appropriate spatial weight function is important for ensuring the correctness of the regression coefficients in the model. So the Gauss function is selected as the spatial weight function in this paper.

12 $${\text{w}}_{\text{ij}}=\text{exp}\left[-{\left(\frac{{\text{d}}_{\text{ij}}}{\text{b}}\right)}^{2}\right]$$

${\text{w}}_{\text{ij}}$ is the weight. b is the bandwidth. ${\text{d}}_{\text{ij}}$ is the distance between position i in space and position j in space.

Using Bowman's method, b is related to CV as follows:

13 $$CV=\sum_{i=1}^{n}{\left[{y}_{i}-{y}_{\ne i}\left(b\right)\right]}^{2}$$

${\text{y}}_{\ne \text{i}}\left(\text{b}\right)$ is the fitted value of ${\text{y}}_{\text{i}}$. When the CV is the smallest, the corresponding b is the corresponding bandwidth.

### Data source

The study subjects includes 30 administrative units, comprising provinces, cities and autonomous regions with the exclusion of Hong Kong, Macao, Taiwan and Tibetan areas. The data used for the indicators in this paper were obtained from Statistical Yearbook^30–37^ and National Bureau of Statistics. Given that this paper applied a geographically weighted regression model to study the spatial variability of the driving factors of forest ecological security, cross-sectional data were examined, and the forest resource data were updated every five years. Therefore, this paper selected a total of two years, 2013 and 2018, as the study time to analyze the spatial variability of the driving factors of interprovincial forest ecological security in these two stages.

## Results

### Analysis of the spatiotemporal pattern of forest ecological security in China

#### Spatiotemporal Distribution of Forest Ecological Security in China

**Figure 1:**
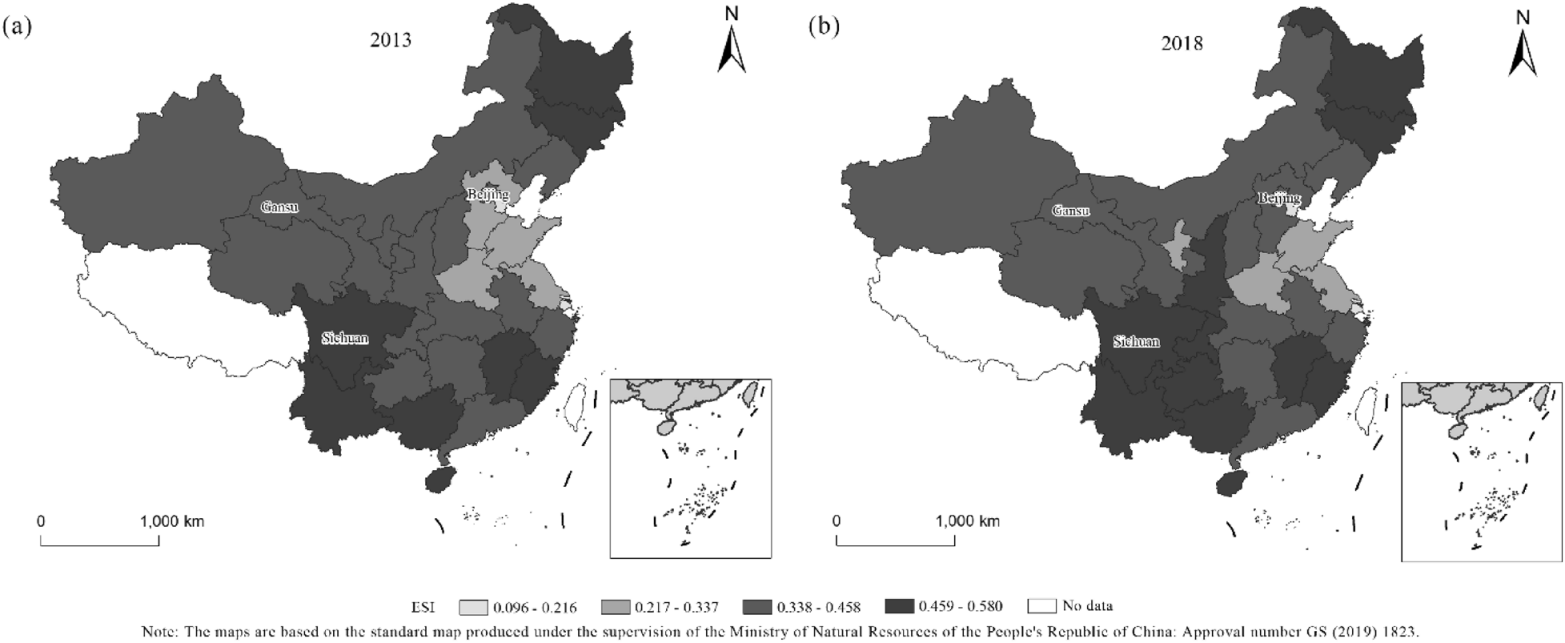
Spatiotemporal distribution of the forest ecological security index (ESI). (a) Description of the spatiotemporal distribution of the forest ecological security index (ESI) in 2013; (b) Description of the spatiotemporal distribution of the forest ecological security index (ESI) in 2018.

In this paper, we refer to the PSR model to construct the framework of the forest ecological security index system and adopt the entropy weight method to calculate the forest ecological security index in 30 provinces, cities and autonomous regions of China. The forest ecological security indexes for 2013 and 2018 are shown in Fig. 1.

First, in terms of the change trend, the average value of China's forest ecological security index increased from 0.400 to 0.411 from 2013 to 2018, which was a relatively small increase. It indicates that the level of China's forest ecological security improved to a small extent from 2013 to 2018. In recent years, China has implemented various measures, such as land greening and forest nurturing, to protect and restore forests, improving the level of forest volume. In 2013, China's forest coverage rate was 21.63%, which increased to 22.08% in 2018, conducive to improving the level of forest ecological security. The improvements in forest ecological security levels in Beijing, Gansu and Sichuan are closely related to the continuous afforestation and strengthening of ecological civilization construction in these areas. In addition, the forest ecological security level in other provinces and cities has not changed (Fig. 1).

Second, in terms of region-wide distribution, the average values of the forest ecological security indexes of China's 30 provinces, cities and autonomous regions were 0.400 and 0.411 in 2013 and 2018, respectively. The overall value of the forest ecological security index is at a general level. Its distribution is in an unbalanced state. This is related to China's vast territory and different natural and geographical conditions, as well as the level of forest protection in various regions. In 2013 and 2018, the provinces and autonomous regions with forest ecological security indexes less than 0.4 were located in the North China Plain region and the northwestern region. The former has a vast plain area, high population density, early forest development and utilization and less precipitation. In comparison, the northwestern region is more arid and has more harsh natural conditions, which are not conducive to the improvement of forest ecological security in the above-mentioned areas. The value in the central region ranges from 0.4 to 0.5, indicating relatively good natural and geographical conditions. In recent years, the ecological protection has also been strengthened.

**Figure 2:**
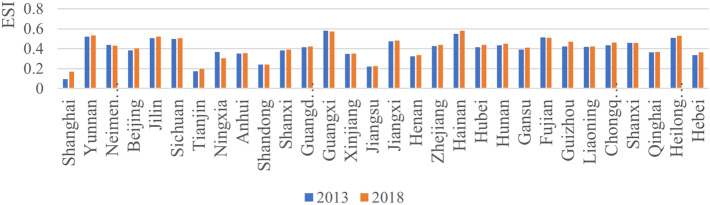
Forest ecological security index of China in 2013 and 2018.

Third, in terms of the values, in 2013, the Guangxi Zhuang Autonomous Region had the highest level of forest ecological security (0.579), and Shanghai had the lowest level (0.096). In 2018, Hainan had the highest level of forest ecological security (0.580), while Shanghai's level remained the lowest (0.169) (Fig. 2). Guangxi and Hainan belong to the subtropical and tropical monsoon climates, respectively, with sufficient heat and abundant precipitation conducive to tree growth. In addition, large-scale afforestation and forest coverage have a high level of ecological security. While Shanghai has a high level of urbanization, dense population, limited urban green land and low forest coverage, which is not conducive to improving forest ecological security.

#### Characteristics of spatial clustering pattern changes in forest ecological security in China

**Figure 3:**
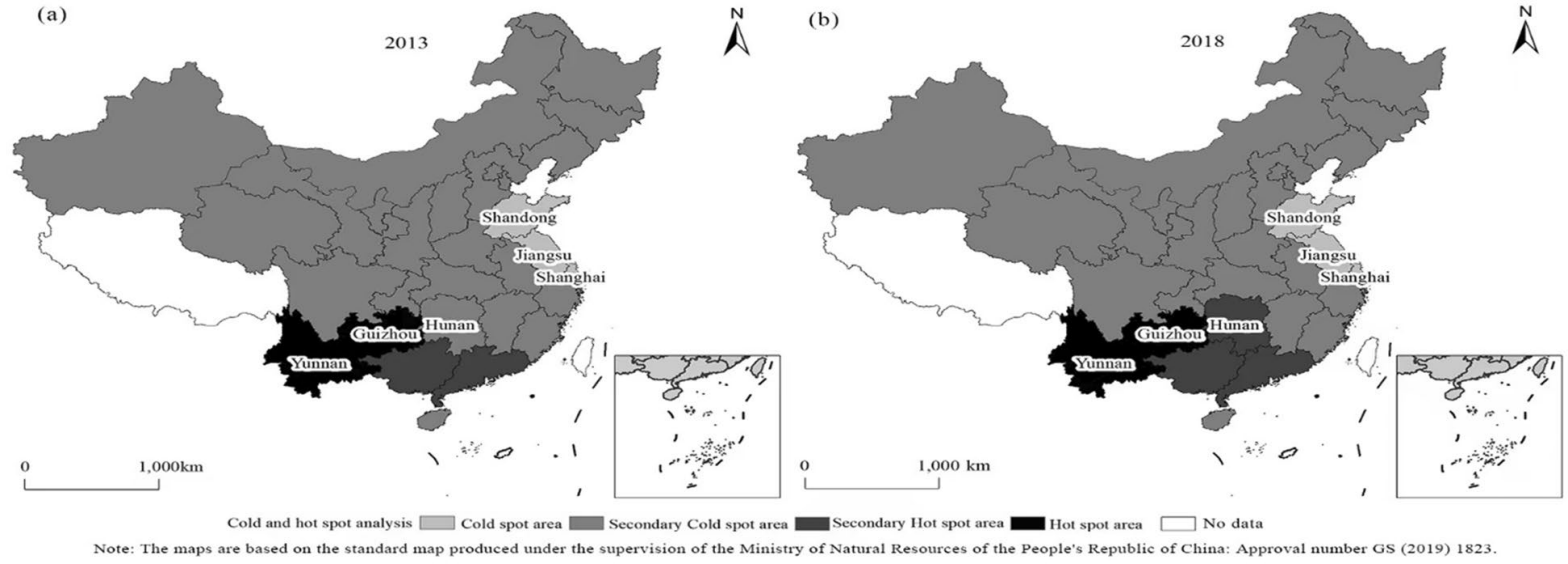
Spatial clustering pattern evolution of forest ecological security in China in 2013 and 2018. (**a**) Description of the spatial clustering pattern of forest ecological security in China in 2013; (**b**) description of the spatial clustering pattern of forest ecological security in China in 2018.

To comprehensively examine the spatial clustering pattern and its changing characteristics in terms of forest ecological security in China, the local spatial autocorrelation method was used to calculate the local correlation index Getis Ord GI * of the spatial distribution. The Jenks best natural fracture method in ArcGIS 10 was used to divide the local GI statistical values into four levels from low to high, namely, cold spot area, secondary cold spot area, secondary hot spot area and hot spot area, and to generate a distribution map of cold and hot spots in China's forest ecological security index (Fig. 3). Figure 3 shows that overall, the distribution of Getis Ord GI values for forest ecological security in China exhibited a clustered pattern, with relatively concentrated distributions in cold and hot areas. From 2013 to 2018, there was little change in the spatial clustering pattern of forest ecological security in China. Specifically, there was no change in cold or hot spot areas. Cold spot areas were concentrated in provinces and cities such as Shandong, Jiangsu and Shanghai. These areas are all located in the northern part of China, which has vast plains and developed agriculture but poor site conditions, low precipitation, a relatively developed economy, a high urbanization level, a dense population and low forest coverage. Although the protection of forest resources has been strengthened in recent years, it is still difficult to reverse the status of forest ecological security. The hot spot areas are mainly concentrated in the Yunnan and Guizhou regions, which are exactly the opposite of the cold spot areas. The terrain is rugged, precipitation is abundant, the temperature is high, and the area is suitable for forest growth. In addition, the level of urbanization is relatively low, the population density is low, and forest destruction is minimal. The secondary cold spot area occupies the vast majority of provinces and cities in China. Hunan transformed from a secondary cold spot area in 2013 to a secondary hot spot area in 2018, indicating an improvement in forest ecological security. At the end of 2013, the forest coverage rate in Hunan was 57.52%. By the end of 2018, the forest coverage rate in Hunan had increased to 59.82%. By eliminating barren mountains and returning farmland to forests, Hunan has achieved afforestation and greening. The implementation of collective forest rights system reform has increased the enthusiasm of foresters for protecting and loving forests, effectively reducing the occurrence of forest disasters, and enabling forest resources to enter a period of rapid growth.

### Empirical results and analysis of the driving factors of forest ecological security

The results of the geographically weighted regression model test for the driving factors of forest ecological security in 2013 and 2018 are shown in Table 3. In the estimation results, R^2^ is a measure of fitting degree. Its value varies from 0.0 to 1.0, the bigger the better. The smaller the Residual Squares is, the better the GWR model fits the observed data. AICc helps to compare different regression models. Models with lower AICc values will fit the observed data better. So the overall structure of this model is feasible. The fitting degree in 2018 is higher than in 2013.

**Table 3:** All the estimation results of the GWR model.

Indicators	2013	2018
R^2^	0.382	0.459
R^2^ Adjusted	0.275	0.315
Residual squares	0.926	1.046
Bandwidth	2,915,615.462	1,872,116.891
AICc	− 4.501	2.910

**Figure 4:**
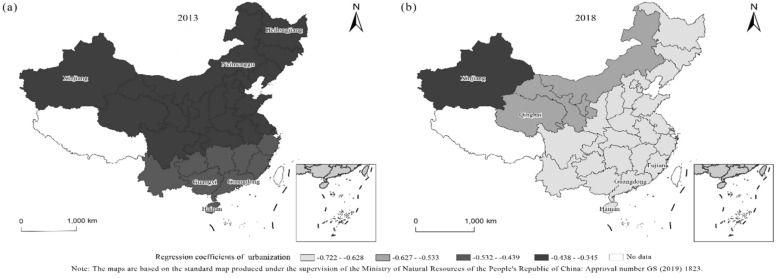
Regression coefficients of urbanization in 2013 and 2018. (**a**) Description of regression coefficients of urbanization in 2013. (**b**) Description of regression coefficients of urbanization in 2018.

**Figure 5:**
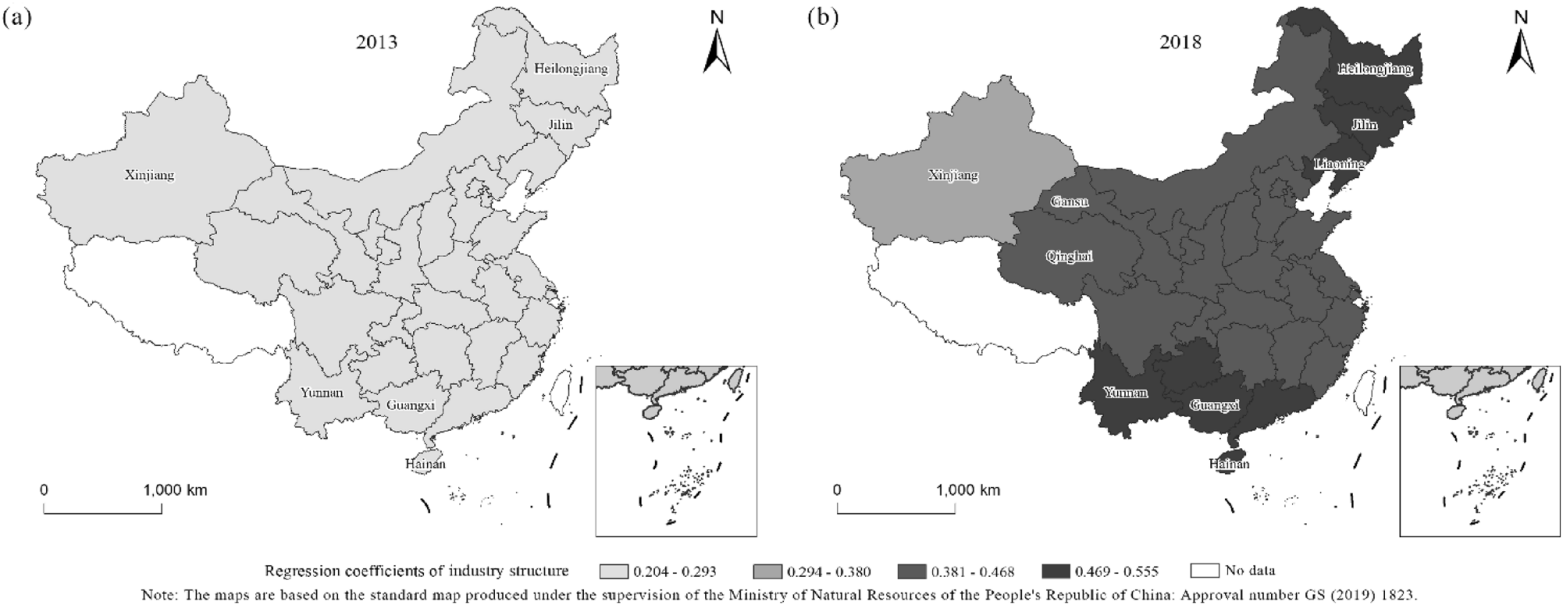
Regression coefficients of industry structure for two years. (**a**) Description of regression coefficients of industry structure in 2013. (**b**) Description of regression coefficients of industry structure in 2018.

**Figure 6:**
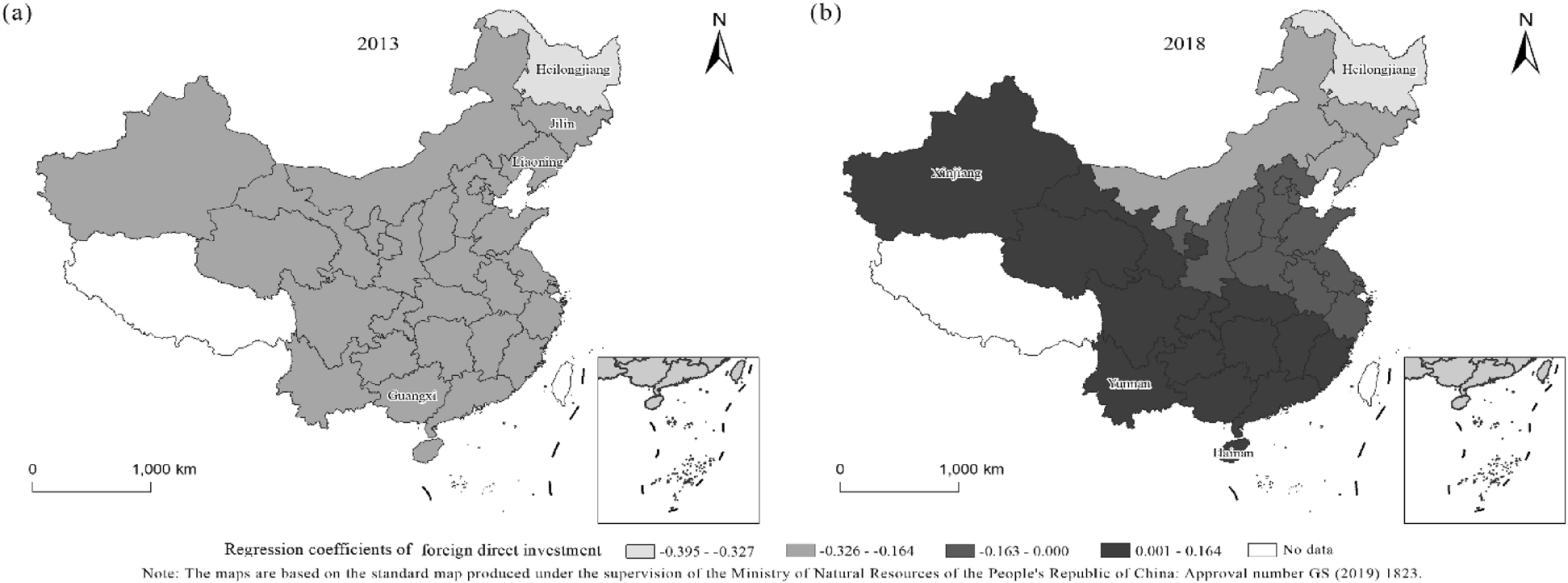
Regression coefficients of FDI for two years (**a**) Description of regression coefficients of FDI in 2013; (**b**) Description of regression coefficients of FDI in 2018.

The geographically weighted regression results for 2013 and 2018 are shown in Tables 4, 5. Using the geographically weighted regression model, the regression equation can be obtained for each province, city and autonomous region. Since the regression model considers the interaction between the spatial geographic locations of each province, city and autonomous region, the results can be used to analyze the spatial heterogeneity of them. The article analyses the influence degree of each factor in combination with the regression results (Tables 4, 5 and Figs. 4, 5, 6). It finds that the level of urbanization has a greater influence on forest ecological security than the industrial structure and FDI. The industrial structure had a positive impact on forest ecological security in 2013 and 2018. In 2018, foreign direct investment was positively correlated with forest ecological security in 14 provinces, while it was negatively correlated in other provinces. In 2013, foreign direct investment was negatively correlated with forest ecological security in all provinces. The level of urbanization was negatively correlated with forest ecological security in both 2013 and 2018. The following is a detailed analysis of the impact of each variable on forest ecological security in each province, city, and autonomous region.

**Table 4:** The results of GWR in 2013.

Region	Fitting coefficient	Constant term	Industrial structure	Foreign direct investment	Urbanization level	Residual	Standard error
Shanghai	0.370	0.785	0.242	− 0.245	− 0.433	− 0.265	0.156
Yunnan	0.327	0.773	0.281	− 0.167	− 0.448	0.090	0.177
Neimenggu	0.337	0.758	0.219	− 0.277	− 0.372	0.084	0.183
Beijing	0.349	0.767	0.227	− 0.268	− 0.393	0.040	0.070
Jilin	0.364	0.773	0.214	− 0.308	− 0.380	0.169	0.185
Sichuan	0.326	0.765	0.261	− 0.192	− 0.426	0.128	0.186
Tianjin	0.352	0.769	0.229	− 0.266	− 0.397	− 0.256	0.149
Ningxia	0.328	0.759	0.239	− 0.229	− 0.399	− 0.114	0.187
Anhui	0.361	0.780	0.246	− 0.236	− 0.430	− 0.166	0.187
Shandong	0.358	0.774	0.235	− 0.256	− 0.411	− 0.300	0.188
Shanxi	0.343	0.766	0.236	− 0.246	− 0.403	− 0.044	0.186
Guangdong	0.360	0.790	0.271	− 0.195	− 0.461	0.253	0.158
Guangxi	0.349	0.784	0.277	− 0.183	− 0.457	0.245	0.184
Xinjiang	0.253	0.721	0.218	− 0.197	− 0.345	− 0.181	0.177
Jiangsu	0.364	0.781	0.241	− 0.246	− 0.426	− 0.081	0.120
Jiangxi	0.362	0.785	0.257	− 0.216	− 0.446	0.112	0.186
Henan	0.351	0.773	0.245	− 0.234	− 0.420	− 0.239	0.186
Zhejiang	0.370	0.787	0.248	− 0.234	− 0.441	0.183	0.186
Hainan	0.355	0.792	0.288	− 0.171	− 0.472	0.160	0.155
Hubei	0.351	0.776	0.253	− 0.219	− 0.432	0.027	0.188
Hunan	0.353	0.781	0.262	− 0.205	− 0.445	− 0.004	0.188
Gansu	0.312	0.751	0.238	− 0.219	− 0.391	− 0.147	0.180
Fujian	0.368	0.790	0.258	− 0.217	− 0.453	0.320	0.183
Guizhou	0.341	0.777	0.270	− 0.189	− 0.445	− 0.142	0.175
Liaoning	0.360	0.772	0.221	− 0.288	− 0.390	0.176	0.181
Chongqing	0.341	0.773	0.260	− 0.204	− 0.433	0.093	0.186
Shanxi	0.338	0.766	0.244	− 0.227	− 0.412	0.096	0.186
Qinghai	0.299	0.748	0.245	− 0.197	− 0.395	− 0.151	0.185
Heilongjiang	0.361	0.770	0.205	− 0.330	− 0.361	0.229	0.178
Hebei	0.349	0.767	0.229	− 0.264	− 0.396	− 0.185	0.187

**Table 5:** The results of GWR in 2018.

Region	fitting coefficient	Constant term	Industrial structure	Foreign direct investment	Urbanization level	Residual	Standard error
Shanghai	0.359	0.760	0.438	− 0.038	− 0.694	− 0.213	0.166
Yunnan	0.328	0.744	0.523	0.094	− 0.668	0.064	0.187
Neimenggu	0.331	0.706	0.467	− 0.181	− 0.587	0.117	0.197
Beijing	0.344	0.723	0.467	− 0.132	− 0.628	0.020	0.060
Jilin	0.400	0.756	0.521	− 0.288	− 0.652	0.195	0.195
Sichuan	0.310	0.723	0.463	0.064	− 0.645	0.115	0.202
Tianjin	0.347	0.727	0.466	− 0.123	− 0.637	− 0.167	0.150
Ningxia	0.304	0.703	0.436	− 0.018	− 0.608	− 0.232	0.202
Anhui	0.350	0.750	0.445	− 0.018	− 0.685	− 0.204	0.204
Shandong	0.349	0.737	0.457	− 0.082	− 0.658	− 0.356	0.206
Shanxi	0.329	0.718	0.450	− 0.059	− 0.632	− 0.035	0.205
Guangdong	0.363	0.781	0.472	0.062	− 0.722	0.141	0.073
Guangxi	0.354	0.769	0.497	0.075	− 0.705	0.194	0.196
Xinjiang	0.139	0.598	0.322	0.096	− 0.407	− 0.156	0.169
Jiangsu	0.354	0.750	0.446	− 0.045	− 0.681	− 0.241	0.178
Jiangxi	0.355	0.766	0.446	0.029	− 0.707	0.129	0.203
Henan	0.339	0.735	0.448	− 0.019	− 0.663	− 0.275	0.202
Zhejiang	0.359	0.766	0.434	− 0.009	− 0.704	0.231	0.199
Hainan	0.373	0.788	0.533	0.081	− 0.717	0.178	0.139
Hubei	0.341	0.745	0.450	0.018	− 0.679	0.090	0.206
Hunan	0.348	0.759	0.461	0.047	− 0.698	0.017	0.206
Gansu	0.274	0.683	0.419	0.005	− 0.572	− 0.163	0.182
Fujian	0.361	0.777	0.438	0.028	− 0.718	0.366	0.192
Guizhou	0.339	0.752	0.483	0.069	− 0.686	− 0.080	0.193
Liaoning	0.373	0.741	0.492	− 0.205	− 0.645	0.187	0.201
Chongqing	0.331	0.740	0.460	0.046	− 0.671	0.227	0.197
Shanxi	0.321	0.719	0.445	− 0.007	− 0.637	0.147	0.204
Qinghai	0.250	0.676	0.416	0.059	− 0.561	− 0.133	0.199
Heilongjiang	0.427	0.772	0.555	− 0.395	− 0.653	0.202	0.186
Hebei	0.343	0.723	0.464	− 0.118	− 0.631	− 0.142	0.204

The regression coefficients of urbanization in 2013 and 2018 (Fig. 4) show that.

- First, the impact of urbanization on forest ecological security has a significant spatial dependence effect. For example, in 2013, the urbanization regression coefficients of the three northeastern provinces were all between − 0.361 and − 0.393, while the coefficients of Guangdong, Guangxi and Hainan, which belong to southern China, were between − 0.472 and − 0.457, revealing significant contiguous spatial characteristics in terms of spatial distribution.
- Second, the impact of urbanization level on forest ecological security exhibited negative correlation driving characteristics. In 2013 and 2018, the impact coefficients of urbanization on forest ecological security in each province and region were less than 0. It indicates that urbanization has a negative relationship with forest ecological security.
- Third, from 2013 to 2018, the impact degree of urbanization on forest ecological security increased. The mean value of the impact coefficient in 2013 was − 0.417, while it became − 0.652 in 2018. In 2013, the provinces where urbanization had a greater impact on forest ecological security were mainly located in Hainan (-0.472), Guangdong (-0.461) and Guangxi (-0.457).The provinces where urbanization had a less impact were mainly in Neimenggu (-0.372), Heilongjiang (-0.361) and Xinjiang (-0.345). In 2018, as the impact of urbanization became greater, the regression coefficient of the impact of urbanization on forest ecological security in each province of China became greater than 0.4. Among them, Guangdong (-0.722), Fujian (-0.718) and Hainan (-0.717) had the highest impact degree of urbanization, while Gansu (-0.572), Qinghai (-0.561) and Xinjiang (-0.407) had the lowest impact degree of urbanization. In 2013, the impact of urbanization increased continuously from north to south, while it shifted from the central and western regions to the east in 2018.

The industry structure regression coefficients for 2013 and 2018 (Fig. 5) show that.

- First, the impact of the three-to-two industry ratio on forest ecological security exhibited positive driving characteristics. The impact degree of the three-to-two industry ratio on forest ecological security increased from 2013 to 2018.
- Second, in 2013, the regions with greater impacts of the three-to-two industry ratio on forest ecological security were concentrated in Hainan (0.288), Yunnan (0.281) and Guangxi (0.277). The provinces with smaller impacts were Xinjiang (0.218), Jilin (0.214) and Heilongjiang (0.205). In 2018, provinces such as Heilongjiang (0.556), Hainan (0.533), Yunnan (0.523), Jilin (0.521), Guangxi (0.497) and Liaoning (0.492) had greater impacts on forest ecological security, while Gansu (0.419), Qinghai (0.416) and Xinjiang (0.322) had smaller impacts.
- Third, comparing the regression coefficients of the three-to-two industry ratio between 2013 and 2018, the impact of the three-to-two industry ratio on forest ecological security gradually shifted to the north; in particular, the three northeastern provinces had the largest increase in the elasticity coefficient.

The regression coefficients of FDI in 2013 and 2018 (Fig. 6) show that.

- First, foreign direct investment was negatively correlated with the forest ecological security index in 2013, which proves that the "pollution paradise" hypothesis is valid. Foreign direct investment had a positive effect on the forest ecological security index in 2018 in 16 provinces, cities and autonomous regions, while it had a negative effect on other provinces. A negative correlation indicates that the introduction of FDI is gradually improving the ecological environment of China.
- Second, in 2013, foreign direct investment in Heilongjiang (-0.330), Jilin (-0.308) and Liaoning (-0.288) had the greatest negative impact on forest ecological security, while that in Yunnan (-0.167), Hainan (-0.171) and Guangxi (-0.183) had the least negative impact. In 2018, except for Heilongjiang(-0.395), where the negative impact of FDI on forest ecological security further increased, the impact in all the other regions decreased. The positive impacts of foreign investment on forest ecological security are mainly concentrated in the southern and western regions of China, among which the positive impacts are the greatest in Xinjiang (0.096), Yunnan (0.094), and Hainan (0.081).

## Discussion

In terms of the spatiotemporal evolution of forest ecological security in China, based on the Forest Ecological Security Index in 2013 and 2018, the areas with the best forest ecological security conditions were distributed in the Northeast Forest Region, Southwest Forest Region and Fujian Province. The status of forest ecological security shows a certain degree of spatial agglomeration, which may be related to the natural geographical conditions of the region. For example, Yunnan Province, where the Tropic of Cancer runs through its southern region, has numerous water systems and abundant plants, such as tropical, subtropical, temperate and cold temperate plant types, with a forest coverage rate of 65.04%. Similarly, the status of forest ecological security in Fujian Province is also closely related to its physical and geographical conditions^38^. However, the status of forest ecological security in Northwest China and the North China Plain is poor, and the status in Central China is general. In addition to physical and geographical conditions, forest ecological protection has a significant direct impact on forest ecological security. Compared with those in other regions, the enthusiasm for activities of forest ecological protection in the west is not high^13^. However, overall, the forest ecological security index in China has not changed much, which is consistent with the conclusions of some provinces in China. For example, from 1999 to 2014, the forest ecological security level in Hubei Province exhibited a good overall situation and a stable trend^5^ with relatively small change. Although the level of forest ecosystem security in Shanghai has improved, the values of the index is still at the lowest point. Relevant departments still need to step up ecological construction efforts and continue to improve the level of forest ecological security. The status of forest ecological security the above mentioned is closely related to the natural conditions and stages of economic and social development in the region, especially the stages of industrialization and urbanization and the degree of openness to the outside world.

Among the driving factors of forest ecological security, firstly, the impact of urbanization level on forest ecological security is generally higher than that of industrial structure and foreign direct investment, and there is a negative correlation between urbanization level and forest ecological security. There are two main reasons for this: on the one hand, population agglomeration leads to urban expansion, increasing demand for infrastructure construction, and promoting the vigorous development of the real estate industry, thereby occupying forest ecological land and devouring green space. On the other hand, the extensive urbanization approach leads to an increase in waste emissions and low resource utilization efficiency, which exceeds the carrying capacity of forest ecosystems and puts greater pressure on their self-regulation, reducing the level of forest ecological security. Moreover, this impact continues to increase, which is consistent with the findings of previous research^16,17^. Due to the high level of urbanization in the eastern region and high urban population density, resource consumption and household waste have increased. Urban sprawl, accompanied by changes in land use, has reduced the area of forestry land and had a negative impact on the forest ecological environment. In 2022, China's urbanization rate was 65.22%; although there was a certain gap with the developed countries, it reached the mid-late stage of urbanization. Therefore, in the process of urbanization, more attention should be paid to the protection of forest ecological resources and the promotion of high-quality development.

Second, the industrial structure has a positive impact on forest ecological security. In 2013, the ratio of tertiary to secondary industries in China was 1.05, and the added value of tertiary industry exceeded that of secondary industry for the first time. Tertiary industry itself is an environmentally friendly industry that has a positive impact on forest ecological security. In 2018, the ratio reached 1.28. An increase in the ratio can reduce resource consumption and pollutant emissions. Thus, the level of forest ecological security can be improved. It indicates that upgrading the industrial structure is an effective means to improve forest ecological security. The main reason why the impact of China's industrial structure on forest ecological security has shifted from the south to the north is twofold. First, the industrial structure in the southern region has been adjusted to a relatively high level, which has weakened its impact on forest ecological security. Second, although the economic growth rate in the northeast region has declined, due to the implementation of the Northeast revitalization policy, the tertiary industry has achieved advanced development. The industrial structure has advanced to a certain extent, reducing pressure on forest ecosystems and improving ecological security. However, overall, the proportions of tertiary and secondary industries are positively correlated with the forest ecological security index. Upgrading the industrial structure leads to improvements in forest ecological security^15^. In this regard, the research conclusions are consistent.

Once again, in terms of foreign direct investment, to attract more foreign investment, China often relaxed its environmental control standards and allowed production pollution-intensive and resource-consuming enterprises to move in before. So China became a "pollution paradise" for foreign investment countries. Later, China raised its environmental access standards for foreign investment and tended to choose enterprises with more advanced production technologies and advanced pollution emission management systems. Moreover, this approach can promote regional industrial structure upgrading, reduce the increase in pollutant emissions and improve resource utilization efficiency. Its impact on forest ecological security is gradually developing in a positive direction. But in some provinces, it still shows a negative correlation with forest ecological security. In this regard, existing research has not reached a completely consistent conclusion^18–20^. In fact, based on international research, there are two completely different hypotheses regarding the environmental effects of foreign direct investment. One is the "Pollution Paradise Hypothesis^39^" and the other is the "Pollution Halo Hypothesis^40^". However, this study shows that in China, "Pollution Paradise" and "Pollution Halo" coexist in different provinces. From a dynamic perspective, some provinces have a U-shaped relationship between foreign direct investment and forest ecological security. A possible reason is that the role of FDI in the economic development of different provinces varies in reality, leading to different levels of local government access and supervision. As a result, its relationship with forest ecological security is not the same. Therefore, more attention should be paid to the role of government policies in FDI because environmental regulations have a certain regulatory effect on FDI, which can optimize the inflow quality of FDI^18^. This is also a policy framework component of China's ecological civilization construction and has a positive effect on promoting the improvement of the status of forest ecological security.

In future research, if data for 2023 can be obtained for spatiotemporal evolution analysis of forest ecological security, its development trend will be more clearly presented. In addition, with the further development of the social economy, whether there are more extensive indicators in the PSR model for measuring forest ecological security is an important issue that needs further research. In terms of the choice of method, further in-depth research is needed to determine whether multiple methods can be combined to jointly measure the driving factors of forest ecological security.

## Conclusions

The forest ecological security indexes of 30 provinces, cities, and autonomous regions in China were calculated based on the PSR model and entropy weight method, and the spatiotemporal evolution was analyzed via ArcGIS software. Then, from the perspectives of urbanization, industrial structure, and foreign direct investment, a geographically weighted regression model was used to empirically study the spatial differences in three variables (urbanization, industrial structure, and foreign direct investment) on the forest ecological security index in 2013 and 2018, to investigate the spatial variability in the impact of each driving factor on forest ecological security and to propose corresponding policy suggestions for improving forest ecological security in China.

First, the average values of forest ecological security in China in 2013 and 2018 were 0.39 and 0.41 respectively, which are the general level with a small improvement. It indicates that China still needs to increase its ecological construction efforts and continue to improve its forest ecological security. The distribution of forests in China is uneven. The best forest ecological security areas are distributed in the northeast forest region, southwest forest region and Fujian, while the worst are located in the North China Plain region and the northwest region.

Second, the impacts of urbanization, industrial structure and foreign direct investment on forest ecological security have significant spatial differences, and there is an obvious spatial dependence. The government should develop synergistic plans for neighboring provinces to improve forest ecological security. However, different provinces have different impact effects and spatial variabilities. Therefore, the government should seek the best path to enhancing forest ecological security in each province, city, and autonomous region according to the characteristics of different regions when drawing the red line of forest ecological security and formulate differentiated and effective policies according to local conditions.

Third, urbanization has the greatest impact on forest ecological security in each province, city, and autonomous region. From 2013 to 2018, the impact of urbanization on forest ecological security has been increasing in each province, city and autonomous region, especially in the southern region. On the one hand, all regions continue to promote rural revitalization, increase the income level of farmers, narrow the income gap between urban and rural areas, slow down the pace of urbanization, and promote high-quality urbanization; on the other hand, they plan urban layouts rationally, control the further spatial expansion of large cities, and improve the intensification of urban construction land.

Fourth, the impact of industrial structure on forest ecological security shows positive driving characteristics. From 2013 to2018, the degree of impact of the three-to-two industry ratio on forest ecological security has increased, indicating that industrial structure upgrading is an effective way to improve forest ecological security. From a regional perspective, the impact is greater in northern China and southwest China. On the one hand, we should continue to optimize the industrial structure and increase the proportion of tertiary industry, especially resource-dependent and energy-dependent industries, and develop industrial system construction strategies to avoid "one size fits all". On the other hand, the adjustment of industrial structure should involve precise, effective use of the advantages of the regional resource endowment and actively explore industries with provincial characteristics. For example, in the three northeastern provinces and the west, green and modern agriculture and tourism should be developed, featuring forests, borders, ice, and snow.

Fifth, foreign direct investment was negatively correlated with forest ecological security in 2013, indicating that there was a "pollution paradise effect" of foreign direct investment. Fourteen provinces, cities, and autonomous regions had a positive impact on the forest ecological security index in 2018, mainly in southern and western China, while other provinces had a negative correlation. This indicates that the "pollution paradise effect" of FDI has weakened, while the "pollution paradise" still exists in the eastern and northern regions. The spatial differences among provinces, cities, and autonomous regions should be taken into account; for the eastern and northern regions, economic development should not make them FDI pollution refuges; barriers to entry for FDI should be appropriately raised and we will introduce technology spillover and environmental protection-oriented enterprises. For existing FDI enterprises, we should strengthen environmental protection supervision and improve energy conservation, emission reduction and environ-mental technology in foreign-funded enterprises.

## Data Availability

The datasets used and/or analyzed during the current study available from the corresponding author on reasonable request.
